# Comparison of coconut water and a carbohydrate-electrolyte sport drink on measures of hydration and physical performance in exercise-trained men

**DOI:** 10.1186/1550-2783-9-1

**Published:** 2012-01-18

**Authors:** Douglas S Kalman, Samantha Feldman, Diane R Krieger, Richard J Bloomer

**Affiliations:** 1 Miami Research Associates, Nutrition/Endocrinology Department, 6141 Sunset Drive, Suite 301, Miami, FL 33143; 2 The University of Memphis, Cardiorespiratory/Metabolic Laboratory, Department of Health and Sport Sciences, 106 Roane Fieldhouse, Memphis, TN 38152

**Keywords:** Hydration, Coconut Water, Sport Drink, Exercise

## Abstract

**Background:**

Sport drinks are ubiquitous within the recreational and competitive fitness and sporting world. Most are manufactured and artificially flavored carbohydrate-electrolyte beverages. Recently, attention has been given to coconut water, a natural alternative to manufactured sport drinks, with initial evidence indicating efficacy with regard to maintaining hydration. We compared coconut water and a carbohydrate-electrolyte sport drink on measures of hydration and physical performance in exercise-trained men.

**Methods:**

Following a 60-minute bout of dehydrating treadmill exercise, 12 exercise-trained men (26.6 ± 5.7 yrs) received bottled water (BW), pure coconut water (VitaCoco^®^: CW), coconut water from concentrate (CWC), or a carbohydrate-electrolyte sport drink (SD) [a fluid amount based on body mass loss during the dehydrating exercise] on four occasions (separated by at least 5 days) in a random order, single blind (subject and not investigators), cross-over design. Hydration status (body mass, fluid retention, plasma osmolality, urine specific gravity) and performance (treadmill time to exhaustion; assessed after rehydration) were determined during the recovery period. Subjective measures of thirst, bloatedness, refreshed, stomach upset, and tiredness were also determined using a 5-point visual analog scale.

**Results:**

Subjects lost approximately 1.7 kg (~2% of body mass) during the dehydrating exercise and regained this amount in a relatively similar manner following consumption of all conditions. No differences were noted between coconut water (CW or CWC) and SD for any measures of fluid retention (p > 0.05). Regarding exercise performance, no significant difference (p > 0.05) was noted between BW (11.9 ± 5.9 min), CW (12.3 ± 5.8 min), CWC (11.9 ± 6.0 min), and SD (12.8 ± 4.9 min). In general, subjects reported feeling more bloated and experienced greater stomach upset with the CW and CWC conditions.

**Conclusion:**

All tested beverages are capable of promoting rehydration and supporting subsequent exercise. Little difference is noted between the four tested conditions with regard to markers of hydration or exercise performance in a sample of young, healthy men. Additional study inclusive of a more demanding dehydration protocol, as well as a time trial test as the measure of exercise performance, may more specifically determine the efficacy of these beverages on enhancing hydration and performance following dehydrating exercise.

## Background

Fluid loss during strenuous, long duration exercise is commonplace and can result in thermal stress, impaired cognition and cardiovascular function, accelerated fatigue, and impaired exercise performance [1,2]. Recommendations for fluid intake before, during, and following exercise are well described [3,4] and are typically followed by most athletes seeking enhanced physical performance. Abiding by such recommendations appears particularly important when exercising in hot and humid environmental conditions, where fluid loss may be high [5].

Although water is often suggested to many general fitness enthusiasts who may exercise for relatively short periods of time ( < 75 minutes), carbohydrate-electrolyte sport drinks are highly recommended and appear to be the beverage of choice for most serious athletes--aerobic athletes in particular [2]. This is partly fueled by scientific recommendations for the consumption of such beverages [6,7], and partly by the widespread marketing campaigns of large sport nutrition and beverage companies. Regardless, carbohydrate-electrolyte beverages are widely consumed and represent a multi-billion dollar segment of the food and beverage industry [8].

Some individuals prefer natural alternatives to the manufactured sport drinks. For example, many sport drinks contain fructose and/or maltodextrin, artificial flavors and sweeteners, and added electrolytes (e.g., sodium, potassium). With more emphasis recently within the sport nutrition industry on "natural" beverages, some athletes and recreationally active fitness enthusiasts seek alternatives to the manufactured sport drink. While water is indeed a reasonable solution for some, those involved in longer duration exercise bouts often require and demand carbohydrate and electrolytes within their beverage of choice. For these individuals, coconut water may be considered as one viable alternative.

Coconut water is naturally occurring, is very rich in potassium, contains sodium, chloride, and carbohydrate [9], and is viewed as the hydrating beverage of choice in certain parts of the world [10]. Clinically, coconut water may be used as an oral rehydration aid to replace fluid loss from the gastrointestinal tract in patients suffering severe dehydration due to diarrhea [11,12]. It has also been used intravenously with success [13]. Although not linked specifically to hydration, coconut water has been reported to have antioxidant properties [14], which may aid in neutralizing reactive oxygen species production resulting from long duration exercise [15].

In relation to sport nutrition, coconut water has been reported to provide hydrating effects similar to those of carbohydrate-electrolyte sport drinks [16-18]. Unfortunately, these studies have focused exclusively on hydration measures as primary outcome variables (following a period of dehydrating exercise and consumption of the assigned beverage), while not emphasizing actual exercise performance during the rehydrating period. Hence, while the rehydrating effects of coconut water may be similar to those of carbohydrate-electrolyte sport drinks, an equally important question for most athletes and coaches is whether or not the hydration status equates to actual physical performance. Considering the above, we investigated the effects of two different forms of coconut water (concentrated and not from concentrate) and a carbohydrate-electrolyte sport drink on measures of hydration status and physical performance in exercise-trained men.

## Methods

### Subjects and Screening

Exercise-trained men were recruited to participate. Eligibility was determined by completion of a health history form (Physical Activity Readiness Questionnaire [PAR-Q]) and physical examination. Prior to the start of the study, subjects were engaged in a program of regular exercise for a minimum of the past six months, without difficulty in walking or running on a treadmill. All subjects were instructed to maintain their pre-study exercise program throughout the course of the study, with the exception of refraining from exercise during the 24 hours prior to each test day. Subjects were nonsmokers, did not report any history of cardiovascular, metabolic, neurological, or orthopedic disorders that may have impacted their ability to participate in the study, and did not start the use of any new nutritional supplement over the course of the study; however, they were allowed to continue using nutritional supplements they had been using prior to beginning the study (e.g., multivitamins). Prior to participation, each subject was informed of all procedures, potential risks, and benefits associated with the study through both verbal and written form in accordance with the approved procedures of the Aspire Institutional Review Board for Human Subjects Research (La Mesa, CA; approval date of December 23, 2010). Subjects signed an informed consent form prior to being admitted into the study.

At the initial screening visits, subjects' height via stadiometer (Holtain Limited; Britain) and body mass via digital scale (Detecto; Webb City, MO) were measured and recorded. Body mass was obtained with subjects wearing only a gown and their underwear. Body mass measures following exercise were obtained only after subjects were thoroughly towel dried. Heart rate and blood pressure (using subjects' left arm) were recorded following a minimum of five minutes of quiet rest, while subjects were seated in a chair. A 12-lead electrocardiogram was obtained and analyzed for normality, to ensure subject suitability for participation. Blood samples were collected from subjects for routine assessment of clinical chemistry parameters (e.g., metabolic panel and complete blood count). Please see Table 1 for subject descriptive characteristics. A familiarization trial of the exercise performance test was also conducted during the initial laboratory visit. A description of this test is provided below.

**Table 1:** Characteristics of 12 exercise-trained men

Variable	Value
Age at Screening (years)	26.6 ± 5.7 24.0 (21.0 - 35.0)
Ethnicity	
Hispanic	12 (100%)
Total	12 (100%)
Race	12 (100%)
Caucasian	12 (100%)
Total	12 (100%)
Height (cm)	175.4 ± 4.1 175.0 (168.6 - 181.2)
Body Mass at Screening (kg)	77.2 ± 6.3 78.4 (66 - 85.8)
Body Mass Index (kg ∙ m^-2^)	25.1 ± 1.8 26.1 (21.5 - 26.9)
Systolic Blood Pressure (mm Hg)	118.4 ± 13.2 120.5 (97.0 - 145.0)
Diastolic Blood Pressure (mm Hg)	73.9 ± 6.7 74.0 (64.0 - 87.0)
Heart Rate (beats ∙ minute^-1^)	68.8 ± 14.4 66.5 (48.0 - 99.0)
Glucose (mg ∙ dL^-1^)	92.5 ± 4.0 91.5 (87.0 - 99.0)
Blood Urea Nitrogen (mg ∙ dL^-1^)	15.2 ± 3.0 16.0 (9.0 - 19.0)
Creatinine (mg ∙ dL^-1^)	1.0 ± 0.2 1.0 (0.7 - 1.2)
Alkaline Phosphatase (Units ∙ L^-1^)	82.0 ± 41.0 73.0 (32.0 - 177.0)
Aspartate Amino Transaminase (Units ∙ L^-1^)	21.4 ± 4.4 20.5 (16.0 - 29.0)
Alanine Amino Transferase (Units ∙ L^-1^)	20.8 ± 5.8 21.0 (11.0 - 30.0)
White Blood Cell count (thousands ∙ μL^-1^)	6.9 ± 1.7 6.7 (4.2 - 9.8)
Red Blood Cell count (millions ∙ μL^-1^)	5.3 ± 0.4 5.3 (4.5 - 6.1)
Hemoglobin (g ∙ dL^-1^)	15.0 ± 1.0 15.3 (13.1 - 16.0)
Hematocrit (%)	47.7 ± 3.0 47.9 (42.8 - 52.2)

Data are mean ± SD (top row); median and (range) provided in bottom row

### Test Days

On each of the four test days, subjects reported to the lab in the morning following an overnight fast (no food or beverages other than water were allowed after midnight). The time of day for testing each subject was matched for all subsequent test days ( ± 60 minutes). Subjects were instructed not to exercise or to consume alcohol during the 24 hours prior to each test day, but to consume water liberally up to the time they reported to the lab for testing. Adherence to study instructions was confirmed with all subjects on each day of testing. Following all baseline assessments, subjects were provided with a standardized breakfast consisting of a bagel and one tablespoon of cream cheese. They were also provided with up to 470 mL of water.

### Dehydrating Exercise Test

Sixty minutes following the conclusion of the standardized breakfast, subjects performed the dehydrating exercise test. It should be noted that of the total of 48 test visits (12 subjects × 4 visits), slight deviations in the time from food intake to the start of the dehydrating exercise test were noted for 14 of the tests (i.e., started before or after the set 60 minute time). Specifically, nine tests were conducted within 15 minutes of this time, two tests were conducted within 30 minutes of this time, and three tests were conducted within 45 minutes of this time. We do not believe these deviations significantly influenced the findings; in particular considering that these were equally dispersed among the four conditions.

The dehydrating exercise consisted of two, 30-minute bouts of walking/jogging, interspersed with a 10 minute rest period. Specifically, subjects walked/jogged at 2, 3, 4, 5, 6 and 7 miles per hour on a motorized treadmill, using a grade of 0%. Five minutes of exercise was performed at each speed. Following the initial 30 minutes of exercise, a 10-minute break was allowed, during which time subjects walked around and/or remained seated. Subjects then repeated the above sequence of speeds for an additional 30 minutes of exercise. Hence, a total of 60 minutes of exercise was performed within the 70 minute period. All exercise was performed in a climate controlled room, with an average temperature of 36° Celsius and an average relative humidity of 48%. This dehydrating exercise protocol has been reported to induce a 2 to 3% reduction in body weight [19]. During the three hour period from the end of the dehydrating exercise test to the start of the performance exercise test, subjects were required to rest quietly without food or beverage intake (with the exception of the assigned condition). During this time, as well as during the performance exercise test, subjects remained in a thermoneutral environment (i.e., 22 degrees Celsius).

### Conditions

Within minutes following the conclusion of the dehydrating exercise test (after all measurements were obtained--see Table 2), subjects received their assigned condition (beverage). The study design involved a random order, single blind (subject and not investigators), cross-over assignment to one of the following four conditions: Supermarket brand bottled water, pure coconut water (VitaCoco^®^; New York, NY), coconut water from concentrate, or a carbohydrate-electrolyte sport drink (5-6% carbohydrate solution). The amount of each beverage was determined based on the total amount of body mass lost during the dehydrating exercise protocol using the equation: 1300 mL ∙ kg^-1 ^× kg loss = amount of beverage consumed (mL). This provided a weighted volume amount of beverage equal to approximately 125% of the actual body mass lost. Based on this, the amount of beverage consumed equated to 2159 ± 249 mL for bottled water, 2220 ± 367 mL for VitaCoco^®^, 2253 ± 358 mL for coconut water from concentrate, and 2184 ± 358 mL for the sport drink, with no differences noted between conditions (p > 0.05). Subjects were allowed 60 minutes to consume the entire volume of beverage. Each condition was consumed on a different test day, with a minimum of five days separating test visits.

**Table 2:** Study timeline and outcome measures

Time → Variable ↓	Pre Dehydrating Exercise Test	Immediately Post Dehydrating Exercise Test	1 Hour Post Dehydrating Exercise Test	2 Hours Post Dehydrating Exercise Test	3 Hours Post Dehydrating Exercise Test†	Immediately Post Performance Exercise Test
Body Mass††	X	X*	X**	X	X	
Plasma Osmolality	X	X			X	
Urine Specific Gravity	X	X			X	
Subjective Measures (VAS)		X	X	X	X	
Heart Rate	X	X			X	X
Blood Pressure	X	X			X	X

† The Performance Exercise Test began following this measurement time (total exercise time was recorded)

†† Body Mass was used to calculate fluid retention (as described in the Methods section)

* For determination of fluid volume to consume

** For determination of "baseline" body mass

### Performance Exercise Test

Three hours after the completion of the dehydrating exercise test (and two hours after subjects consumed their assigned condition), a test of physical performance was conducted using a treadmill as previously done [20]. Specifically, subjects began walking on a motorized treadmill at a self-selected comfortable speed (0% grade) for five minutes. At the conclusion of the five-minute period, the actual performance test began. The protocol involved an increase in intensity every three minutes. While the speed of the treadmill remained constant at 4.2 miles per hour throughout the test, the grade increase in the following manner: min 1-3, 0%; min 4-6, 2.5%; min 7-9, 5%; min 10-12, 7.5%; min 13-15, 10%; min 16-18, 12.5%; min 19-21, 15%. Subjects exercised until volitional exhaustion and the total exercise time was recorded. This identical protocol was administered at the screening visit (for familiarization) and on each of the four test day visits. Therefore, we do not believe that there was any significant degree of "learning" involved with this test.

### Outcome Measures

In addition to the measure of total exercise time obtained in the performance test described above, the following variables were used as outcome measures; some of which have been discussed previously [21]. With regard to hydration status, body mass, fluid retention (based on body mass), plasma osmolality, and urine specific gravity were measured. Specifically, for fluid retention based on body mass, it was expected that the administration of test product at the amount prescribed would bring the subject's body mass back to very near its pre-exercise level. The rehydrating efficacy of the test product was indicated by how well this volume of fluid was retained over the two hours following consumption. Therefore, the body mass immediately post beverage consumption (which occurred at 1 hour post dehydrating exercise) was considered the "baseline"; this was subtracted from the body mass values at 2 and 3 hours post dehydrating exercise, and this difference was divided by the mass of beverage that had been consumed, then multiplied by 100 to yield the "percent of rehydrating fluid retained" at 2 and 3 hours. It should be noted that body mass was accurately determined using an electronic scale with subjects dry and wearing only a gown and underwear. In additional, all fluid volumes delivered to subjects were meticulously measured. Our use of body mass was used as a surrogate efficacy indicator of hydration as done previously [22].

Plasma osmolality and urine specific gravity were determined using standard procedures. Osmolality was determined by freezing point depression. Specific gravity was determined using reagent test strips. Although we did not measure urine osmolality, it has been concluded by Armstrong and colleagues that "urine osmolality and urine specific gravity may be used interchangeably to determine hydration status" [23]. Both plasma osmolality and urine specific gravity have been used previously as indicators of hydration status [24], and were obtained prior to the dehydrating exercise test, immediately following the dehydrating exercise test, and prior to the performance exercise test.

With regard to subjective measures, thirst, bloatedness, refreshed, stomach upset, and tiredness were determined using a 5-point visual analog scale. Answers were scaled from 1 to 5 where 1 was the lowest and 5 was the highest score. These were assessed immediately, 60 minutes, 120 minutes, and 180 minutes following the dehydrating exercise test.

Heart rate and blood pressure were measured at the following times: Prior to the dehydrating exercise test, immediately following the dehydrating exercise test, prior to the performance exercise test, and immediately following the performance exercise test. A schematic of the study timeline for all outcome measures is provided in Table 2.

### Physical Activity and Dietary Intake

Subjects were instructed to maintain their normal physical activity throughout the study period, with the exception of refraining from strenuous physical activity during the 24 hours preceding each test day. They were also given specific instructions regarding abstinence from alcohol consumption during the 24 hours immediately preceding the test days. Dietary intake was to be maintained through the study period, with the exception of reporting to the lab in a fasted state on each of the four test days. No food records were maintained in this study, which may be considered by some to be a limitation of this work.

### Statistical Analysis

The sample size was determined based on convenience and a power analysis was carried out to determine the effect sizes that would provide an 80% chance of obtaining a significant result of p ≤ 0.05, when testing the outcome measures using the paired Student t test. Using a sample of 12 subjects, an 18% difference in fluid retention between products would be needed to detect statistical significance. All numerical variables were tested for normality by the Anderson-Darling test. Outcome measures as described within the text above for each variable, at each time point, were analyzed by the paired Student t test. All analyses were performed using "*R*" statistical software (version 2.13.1; *R *Foundation for Statistical Computing). Statistical significance was set at p ≤ 0.05. The data are presented as mean ± SD.

## Results

### Overview and Adverse Effects

All subjects successfully completed all aspects of this study, with the exception of one subject who was unable to consume the volume of coconut water from concentrate in the allotted time. Therefore, the trial for this subject was not included in the analysis (n = 11 for coconut water from concentrate). Very few adverse events were noted and all were characterized as mild (e.g., stomach upset), likely due to the consumption of a high volume of fluid ( > 2 liters) in a relatively short period of time (≤ 60 minutes).

### Performance Data

Regarding treadmill performance, no significant difference (p > 0.05) was noted in total exercise time between bottled water (11.9 ± 5.9 minutes), VitaCoco^® ^(12.3 ± 5.8 minutes), coconut water from concentrate (11.9 ± 6.0 minutes), and sport drink (12.8 ± 4.9 minutes).

### Hydration Data

In regard to body mass, subjects lost approximately 1.7 kg during the dehydrating exercise (~2% of starting body mass), regained this amount in a similar manner following consumption of all conditions, and slowly lost approximately 1 kg over the subsequent two hours (Table 3). However, body mass (p = 0.023) was slightly greater with coconut water from concentrate compared only to bottled water (when expressed as change from pre dehydrating exercise at 3 hours post dehydrating exercise). No other differences were noted between conditions for body mass (p > 0.05). In regard to fluid retention (based on body mass), similar findings were observed (as this measure is influenced by body mass), with greater values for coconut water from concentrate compared only to bottled water (p = 0.041) at 3 hours post dehydrating exercise. At 3 hours post dehydrating exercise (2 hours after rehydration) values were numerically highest for coconut water from concentrate (~52%), lowest for bottled water (~35%), and intermediate for VitaCoco^® ^and sport drink (~40%); although these differences were not statistically significant (p > 0.05). No other differences were noted between conditions for fluid retention (p > 0.05). Data are presented in Table 4. Plasma osmolality displayed similar results as noted for body mass and fluid retention, with greater values for coconut water from concentrate compared only to bottled water (p = 0.049; when expressed as change from pre dehydrating exercise at 3 hours post dehydrating exercise). In general, this measure increased after dehydrating exercise, indicating dehydration of the subjects, and returned toward baseline at 3 hours post dehydrating exercise, indicating rehydration of the subjects. No other differences were noted between conditions for plasma osmolality (p > 0.05). Data are presented in Table 5. No differences were noted between conditions for urine specific gravity, with this measure relatively constant and within the normal range over the measurement period (p > 0.05). Data are presented in Table 6.

**Table 3:** Body mass of exercise-trained men before and after dehydrating exercise

Time	VitaCoco^®^	Sport Drink	Coconut Water From Concentrate	Bottled Water
Pre Dehydrating Exercise	78.5 ± 7.4 79.2 (65.2 - 89.0)	77.8 ± 7.1 78.2 (65.2 - 87.3)	77.5 ± 7.6 75.6 (64.9 - 88.4)	77.8 ± 7.6 78.2 (64.8 - 89.3)

Immediately Post Dehydrating Exercise	76.9 ± 7.2 77.4 (63.9 - 87.2)	76.1 ± 6.8 76.6 (63.8 - 85.3)	75.8 ± 7.5 73.7 (63.3 - 86.5)	76.2 ± 7.4 76.5 (63.5 - 87.4)

1 hour Post Dehydrating Exercise	78.4 ± 7.3 79.0 (65.5 - 88.9)	77.7 ± 7.2 77.8 (65.1 - 87.6)	77.6 ± 7.6 75.5 (65.0 - 88.6)	77.9 ± 7.6 78.1 (64.9 - 90.1)

2 hours Post Dehydrating Exercise	78.1 ± 7.2 78.3 (65.5 - 88.8)	77.4 ± 7.0 77.6 (65.1 - 87.0)	77.3 ± 7.5 75.2 (64.8 - 88)	77.4 ± 7.5 77.6 (64.7 - 88.9)

3 hours Post Dehydrating Exercise*	77.6 ± 6.9 78.0 (65.5 - 87.9)	77.0 ± 6.8 77.2 (64.9 - 86.3)	76.9 ± 7.4 75.0 (64.6 - 87.6)	76.9 ± 7.3 76.9 (64.5 - 88.0)

Data are mean ± SD (top row); median and (range) provided in bottom row

*Coconut water from concentrate greater than bottled water (p = 0.023); when expressed as change from Pre Dehydrating Exercise at 3 hours Post Dehydrating Exercise. No other differences noted (p > 0.05).

**Table 4:** Fluid retention of exercise-trained men before and after dehydrating exercise

Time	VitaCoco^®^	Sport Drink	Coconut Water From Concentrate	Bottled Water
1 hour Post Dehydrating Exercise	73.6 ± 22.1 76.0 (30.9 - 101.5	76.4 ± 21.1 77.9 (37.6 - 101.5)	83.5 ± 9.7 84.0 (67.2 - 101.5)	82.1 ± 22.3 88.0 (42.8 - 115.9)

2 hours Post Dehydrating Exercise	59.6 ± 31.7 71.4 (-3.8 - 99.0)	60.6 ± 19.5 66.8 (28.4 - 90.9)	67.6 ± 13.7 63.0 (37.8 - 85.5)	56.9 ± 26.6 62.1 (0.0 - 95.7)

3 hours Post Dehydrating Exercise*	39.0 ± 37.9 35.7 (-42.2 - 99.0)	40.3 ± 24.9 38.9 (-5.7 - 74.8)	51.7 ± 14.9 46.2 (29.4 - 75.6)	34.7 ± 23.9 32.9 (-10.7 - 65.5)

Data are mean ± SD (top row); median and (range) provided in bottom row

*Coconut water from concentrate greater than bottled water (p = 0.041) at 3 hours Post Dehydrating Exercise. No other differences noted (p > 0.05).

**Table 5:** Plasma osmolality of exercise-trained men before and after dehydrating exercise

Time	VitaCoco^®^	Sport Drink	Coconut Water From Concentrate	Bottled Water
Pre Dehydrating Exercise	289.3 ± 4.5 288.5 (282.0 - 300.0)	291.5 ± 6.0 290.0 (283.0 - 305.0)	293.0 ± 5.6 291.0 (287.0 - 305.0)	292.0 ± 5.0 291.0 (282.0 - 300.0)

Immediately Post Dehydrating Exercise	297.5 ± 5.6 297.5 (290.0 - 309.0)	297.7 ± 8.0 297.0 (289.0 - 320.0)	297.5 ± 6.1 296.0 (289.0 - 309.0)	297.6 ± 4.5 297.5 (290.0 - 305.0)

3 hours Post Dehydrating Exercise*	291.2 ± 6.6 290.0 (285.0 - 310.0)	289.6 ± 5.5 288.0 (283.0 - 304.0)	291.8 ± 5.7 289.0 (286.0 - 306.0)	290.3 ± 5.1 289.5 (284.0 - 302.0)

Data are mean ± SD (top row); median and (range) provided in bottom row

*Coconut water from concentrate greater than bottled water (p = 0.049); when expressed as change from Pre Dehydrating Exercise at 3 hours Post Dehydrating Exercise. No other differences noted (p > 0.05).

**Table 6:** Urine specific gravity of exercise-trained men before and after dehydrating exercise

Time	VitaCoco^®^	Sport Drink	Coconut Water From Concentrate	Bottled Water
Pre Dehydrating Exercise	1.0204 ± 0.0087 1.02 (1.01 - 1.03)	1.0218 ± 0.0096 1.03 (1.00 - 1.032)	1.0217 ± 0.0106 1.03 (1.01 - 1.03)	1.0231 ± 0.0068 1.03 (1.01 - 1.03)

Immediately Post Dehydrating Exercise	1.0158 ± 0.0102 1.02 (1.01 - 1.03)	1.0165 ± 0.0112 1.018 (1.00 - 1.03)	1.0153 ± 0.0098 1.02 (1.00 - 1.03)	1.0161 ± 0.0077 1.02 (1.00 - 1.03)

3 hours Post Dehydrating Exercise	1.0200 ± 0.0098 1.03 (1.01 - 1.03)	1.0060 ± 0.0037 1.01 (1.00 - 1.02)	1.0139 ± 0.0066 1.02 (1.00 - 1.03)	1.0055 ± 0.0022 1.01 (1.00 - 1.01)

Data are mean ± SD (top row); median and (range) provided in bottom row

No differences noted (p > 0.05).

### Subjective Data

All four conditions quenched thirst with no significant differences between conditions (p > 0.05). Subjects reported feeling bloated with all four conditions, as per statistically significant increases at 1 hour post dehydrating exercise. Over the two hour rehydration period, the bloatedness decreased for all four conditions but remained statistically significant at 3 hours post dehydrating exercise for VitaCoco^® ^(p = 0.012) and coconut water from concentrate (p = 0.034). Subjects generally felt refreshed after rehydration, with a statistically significant increase for bottled water over VitaCoco^® ^at 1 hour post dehydrating exercise (p = 0.036). No other differences were noted (p > 0.05). The two coconut-based products tended to produce more stomach upset than bottled water or sport drink, with significant findings at 3 hours post dehydrating exercise for VitaCoco^® ^and sport drink (p = 0.034), VitaCoco^® ^and bottled water (p = 0.046), coconut water from concentrate and sport drink (p = 0.020) and coconut water from concentrate and bottled water (p = 0.020). Tiredness generally tended to decrease immediately post dehydrating exercise, with no significant differences between conditions (p > 0.05). All subjective data are presented in Table 7.

**Table 7:** Subjective ratings of exercise-trained men before and after dehydrating exercise

Time	VitaCoco^®^	Sport Drink	Coconut Water From Concentrate	Bottled Water
*Thirst*				
Immediately Post DHE	4.08 ± 1.16	4.42 ± 0.67	4.45 ± 0.69	4.67 ± 0.65
1 hour Post DHE	1.17 ± 0.58	1.33 ± 0.89	1.36 ± 0.67	1.08 ± 0.29
2 hours Post DHE	1.50 ± 0.52	1.58 ± 0.67	1.45 ± 0.52	1.50 ± 0.67
3 hours Post DHE	1.67 ± 0.65	1.83 ± 0.94	1.45 ± 0.82	1.92 ± 1.00

*Bloatedness*				
Immediately Post DHE	1.33 ± 0.49	1.33 ± 0.65	1.55 ± 1.04	1.33 ± 0.49
1 hour Post DHE	3.58 ± 1.00	3.42 ± 1.24	4.00 ± 1.34	3.08 ± 1.24
2 hours Post DHE	2.75 ± 0.97	1.67 ± 0.65	2.82 ± 1.17	1.50 ± 0.67
3 hours Post DHE	2.33 ± 1.23	1.42 ± 0.67	2.45 ± 1.21	1.25 ± 0.62

*Refreshed*				
Immediately Post DHE	1.92 ± 1.00	2.08 ± 1.24	2.09 ± 1.22	1.67 ± 0.89
1 hour Post DHE	3.25 ± 1.36	3.83 ± 1.27	3.82 ± 1.08	4.17 ± 1.19
2 hours Post DHE	3.33 ± 1.23	3.67 ± 1.23	3.64 ± 1.50	3.58 ± 1.16
3 hours Post DHE	3.17 ± 1.19	3.33 ± 1.15	3.55 ± 1.51	3.50 ± 1.09

*Stomach Upset*				
Immediately Post DHE	1.58 ± 0.79	1.25 ± 0.45	1.00 ± 0.00	1.00 ± 0.00
1 hour Post DHE	2.75 ± 1.29	2.00 ± 1.35	3.18 ± 1.66	1.67 ± 0.89
2 hours Post DHE	3.33 ± 1.23	1.25 ± 0.62	3.09 ± 1.51	1.25 ± 0.45
3 hours Post DHE	2.92 ± 1.31	1.17 ± 0.39	2.55 ± 1.44	1.08 ± 0.29

*Tiredness*				
Immediately Post DHE	3.58 ± 1.00	3.92 ± 0.79	3.82 ± 0.98	4.08 ± 0.79
1 hour Post DHE	2.83 ± 0.83	3.08 ± 0.90	2.64 ± 0.92	2.92 ± 1.00
2 hours Post DHE	2.08 ± 0.90	2.58 ± 0.90	2.36 ± 0.81	2.33 ± 0.98
3 hours Post DHE	2.08 ± 0.90	2.50 ± 1.00	2.18 ± 0.98	2.33 ± 0.78

Data are mean ± SD

*Thirst: *No differences between conditions (p > 0.05).

*Bloatedness: *3 hours Post DHE > Immediately Post DHE for VitaCoco^® ^(p = 0.012) and coconut water from concentrate (p = 0.034)

*Refreshed: *1 hour Post DHE > Immediately Post DHE for bottled water compared to VitaCoco^® ^(p = 0.036).

*Stomach Upset: *Greater stomach upset at 3 hours Post DHE for VitaCoco^® ^compared to sport drink (p = 0.034), VitaCoco^® ^compared to bottled water (p = 0.046), coconut water from concentrate compared to sport drink (p = 0.020) and coconut water from concentrate compared to bottled water (p = 0.020).

*Tiredness: *No differences between conditions (p > 0.05).

DHE = Dehydrating Exercise

### Heart Rate and Blood Pressure Data

No differences were noted between conditions for heart rate, systolic blood pressure, or diastolic blood pressure (p > 0.05). Data for these variables are presented in Table 8.

**Table 8:** Heart rate and blood pressure of exercise-trained men before and after dehydrating exercise

Time	VitaCoco^® ^	Sport Drink	Coconut Water From Concentrate	Bottled Water
*Heart Rate*				
Pre DHE	63.6 ± 8.7	63.3 ± 6.7	64.6 ± 11.6	62.7 ± 6.5
Immediately Post DHE	102.1 ± 19.9	101.8 ± 12.8	103.4 ± 13.0	102.0 ± 18.1
Pre PE	70.2 ± 11.2	71.2 ± 9.8	68.5 ± 9.1	64.2 ± 7.6
Immediately Post PE	86.8 ± 15.0	88.0 ± 17.5	96.1 ± 35.7	84.6 ± 15.2

*Systolic Blood Pressure*				
Pre DHE	122.8 ± 9.6	119.6 ± 9.5	121.0 ± 9.4	122.3 ± 8.4
Immediately Post DHE	109.2 ± 9.6	116.8 ± 12.1	113.6 ± 11.7	112.7 ± 4.3
Pre PE	122.1 ± 9.4	116.7 ± 8.4	120.9 ± 9.3	117.6 ± 8.7
Immediately Post PE	120.8 ± 11.9	121.6 ± 9.6	117.7 ± 9.9	115.7 ± 10.3

*Diastolic Blood Pressure*				
Pre DHE	77.8 ± 5.2	75.0 ± 7.5	78.3 ± 7.3	76.7 ± 3.9
Immediately Post DHE	66.8 ± 7.1	73.1 ± 7.0	71.1 ± 7.3	72.2 ± 5.9
Pre PE	76.3 ± 4.3	74.1 ± 5.6	74.8 ± 5.8	73.5 ± 5.2
Immediately Post PE	77.6 ± 6.6	76.6 ± 6.4	74.5 ± 6.6	74.3 ± 7.5

Data are mean ± SD

No differences noted (p > 0.05).

DHE = Dehydrating Exercise

PE = Performance Exercise

## Discussion

Findings from the present investigation indicate that all of the tested beverages are capable of promoting rehydration after one hour of dehydrating exercise. With few exceptions at selected time points, findings for all rehydration variables were essentially the same when comparing the carbohydrate-electrolyte sport drink, coconut water (concentrated and not from concentrate), and bottled water. Moreover, no differences were noted in treadmill performance during the rehydration period. These data are specific to a sample of young, exercise-trained, healthy men.

Maintaining hydration status is vital for athletes and can directly impact exercise performance [25]. As such, many studies have been conducted to determine the optimal rehydration strategies. While water intake is likely an adequate rehydration approach for many individuals, others (e.g., athletes involved in vigorous training) may require intake of water-carbohydrate or carbohydrate-electrolyte mixtures [2], in addition to other nutrients [26]. Such an approach has been reported to be superior to water alone and is generally considered the ideal recommendation for individuals engaged in long duration, strenuous bouts of acute exercise [2,4].

Related to the above, the use of coconut water has been considered by many, as this beverage provides a natural source of carbohydrate and electrolytes [9]. Specifically, coconut water has been reported to provide sugar (~1 g ∙ dL^-1^), potassium (~51 mEq ∙ L^-1^), sodium (~33 mEq ∙ L^-1^), and chloride (~52 mEq ∙ L^-1^) [9]; however, this may vary depending on species of coconut palm. Coconut water has been reported to provide hydrating effects similar to those of carbohydrate-electrolyte sport drinks [16-18].

Saat and colleagues used a cross-over study to assess the effectiveness of fresh young coconut water and a carbohydrate-electrolyte beverage, compared to water on measures of whole body rehydration and blood volume restoration during a two hour rehydration period following a bout of dehydrating exercise [16]. A sample of eight young men participated and consumed the assigned beverage at a volume equal to 120% of the fluid loss during exercise. No statistically significant differences were noted between conditions for any outcome measure; however, blood volume restoration was noted to be slightly greater for coconut water. This same group reported similar findings in a follow-up study published in 2007 [17], using the same volume of beverages (120% of fluid loss during exercise). More recently, Idárraga and Aragón-Vargas studied the rehydrating effect of coconut water following exercise [18]. On three different days, six men and five women were dehydrated to approximately 2% body mass by exercising in a climate-controlled laboratory. On each day and in random order, subjects were rehydrated with fresh coconut water, a sport drink, or plain water using a volume equal to 120% of body weight lost during exercise. Subjects were then monitored for three hours, with urine collection every 30 minutes. No differences were noted between coconut water and sport drink for urine volume or fluid retention (both were better than plain water).

These above studies focused exclusively on hydration measures, following a period of dehydrating exercise and consumption of the assigned beverage, while not emphasizing exercise performance during the rehydrating period. The present study, using a similar fluid volume as used previously, extends these findings by noting similar exercise performance results for natural coconut water (concentrated and not from concentrate) and a carbohydrate-electrolyte sport drink. For most athletes and coaches, this finding is likely of most importance. Our data indicate that coconut water can provide similar benefits as compared to a typical sport drink in terms of exercise performance (as measured based on treadmill time to exhaustion), in addition to measures of hydration. That being said, one potential concern is subject tolerance to coconut water in such high volumes. Subjects reported feeling somewhat bloated and experienced mild stomach upset with the two forms of coconut water used in the present investigation (Table 7), which is likely due to the high volume of fluid required to be consumed in such a short period of time. As with most beverages, individual tolerance to coconut water should be determined prior to use.

It should be noted that this study explored many endpoints at many time-points, each being compared between four products. Consequently, many hundreds of separate pairwise comparisons were carried out, each generating a p value, raising the issue of multiplicity and inflated Type-1 error. No multiple-test adjustments (Bonferroni or other) were applied - it would have been unrealistic and unproductive to try to establish a study-wide 0.05 alpha level, which would have required impossibly small p-values on individual tests. So it should be kept in mind that each individual p value has a one-in-twenty chance of being nominally significant (p < 0.05) purely from random fluctuations. Conclusions of relative efficacy among the different products should not be based simply on isolated p values, but rather on a consideration of the complete set of data for each endpoint. Likewise, observed values were not simply put into a repeated-measures ANOVA to test for overall changes over time - most endpoints displayed very significant changes at certain time points (such as from baseline to immediately post-dehydrating exercise). It was much more appropriate to examination the changes (and differences in mean changes between products) only over certain physiologically-meaningful intervals, and individual t-tests were much more directly interpretable, and therefore more useful, in assessing these specific changes.

## Conclusion

Considering the above, our data indicate that both coconut water (natural, concentrated and not from concentrate) and bottled water provide similar rehydrating effects as compared to a carbohydrate-electrolyte sport drink. Moreover, none of the beverages impacted treadmill exercise performance differently during the rehydration period. Additional study is needed with consideration for the inclusion of a more demanding dehydration protocol, aimed at reducing body mass beyond the 2% mark obtained in the present investigation may be warranted. Finally, while treadmill time to exhaustion is routinely used in laboratory studies, the use of a time trial test as the measure of exercise performance may be more appropriate. Investigators may consider these suggestions when designing future studies focused on the potential rehydrating ability of coconut water and other beverages.

## Competing interests

Financial support for this work was provided by VitaCoco^® ^Company (New York, NY). The investigators have no direct or indirect interest in VitaCoco^®^. RJB has received research funding or has acted as a consultant to nutraceutical and dietary supplement companies.

## Authors' contributions

DSK, SF, and DRK were responsible for the study design, coordination of the study, and oversight of data collection and analysis. RJB assisted in manuscript preparation. All authors read and approved of the final manuscript.

## References

[B1] Rodriguez NR DiMarco NM Langley S American Dietetic Association Dietetians of Canada American College of Sports Medicine Position of the American Dietetic Association, Dietitians of Canada, and the American College of Sports Medicine: Nutrition and athletic performance J Am Diet Assoc 2009 109 3 509 27 19278045 10.1016/j.jada.2009.01.005

[B2] von Duvillard SP Arciero PJ Tietjen-Smith T Alford K Sports drinks, exercise training, and competition Curr Sports Med Rep 2008 7 4 202 8 18607221 10.1249/JSR.0b013e31817ffa37

[B3] American College of Sports Medicine Sawka MN Burke LM Eichner ER Maughan RJ Montain SJ Stachenfeld NS American College of Sports Medicine position stand. Exercise and fluid replacement Med Sci Sports Exerc 2007 39 2 377 90 10.1249/mss.0b013e31802ca597 17277604

[B4] Von Duvillard SP Braun WA Markofski M Beneke R Leithäuser R Fluids and hydration in prolonged endurance performance Nutrition 2004 20 7-8 651 6 10.1016/j.nut.2004.04.011 15212747

[B5] Convertino VA Armstrong LE Coyle EF Mack GW Sawka MN Senay LC Jr Sherman WM American College of Sports Medicine position stand. Exercise and fluid replacement Med Sci Sports Exerc 1996 28 1 i vii 10.1097/00005768-199610000-00045 9303999

[B6] Rehrer NJ Fluid and electrolyte balance in ultra-endurance sport Sports Med 2001 31 10 701 15 10.2165/00007256-200131100-00001 11547892

[B7] Kreider RB Wilborn CD Taylor L Campbell B Almada AL Collins R Cooke M Earnest CP Greenwood M Kalman DS Kerksick CM Kleiner SM Leutholtz B Lopez H Lowery LM Mendel R Smith A Spano M Wildman R Willoughby DS Ziegenfuss TN Antonio J ISSN exercise & sport nutrition review: research & recommendations J Int Soc Sports Nutr 2010 7 7 10.1186/1550-2783-7-720181066

[B8] http://researchwikis.com/Sports_Drink_Market: Accessed 10/18/11

[B9] Chavalittamrong B Pidatcha P Thavisri U Electrolytes, sugar, calories, osmolarity and pH of beverages and coconut water Southeast Asian J Trop Med Public Health 1982 13 3 427 31 7163850

[B10] Kuberski T Roberts A Linehan B Bryden RN Teburae M Coconut water as a rehydration fluid N Z Med J 1979 90 641 98 100 290921

[B11] Chavalittamrong B Pidatcha P Thavisri U Electrolytes, sugar, calories, osmolarity and pH of beverages and coconut water Southeast Asian J Trop Med Public Health 1982 13 3 427 31 7163850

[B12] Adams W Bratt DE Young coconut water for home rehydration in children with mild gastroenteritis Trop Geogr Med 1992 44 1-2 149 53 1496708

[B13] Campbell-Falck D Thomas T Falck TM Tutuo N Clem K The intravenous use of coconut water Am J Emerg Med 2000 18 1 108 11 10.1016/S0735-6757(00)90062-7 10674546

[B14] Mantena SK Jagadish Badduri SR Siripurapu KB Unnikrishnan MK In vitro evaluation of antioxidant properties of Cocos nucifera Linn. water Nahrung 2003 47 2 126 31 10.1002/food.200390023 12744292

[B15] Fisher-Wellman K Bloomer RJ Acute exercise and oxidative stress: a 30 year history Dyn Med 2009 8 1 10.1186/1476-5918-8-1 19144121 PMC2642810

[B16] Saat M Singh R Sirisinghe RG Nawawi M Rehydration after exercise with fresh young coconut water, carbohydrate-electrolyte beverage and plain water J Physiol Anthropol Appl Human Sci 2002 21 2 93 104 10.2114/jpa.21.93 12056182

[B17] Ismail I Singh R Sirisinghe RG Rehydration with sodium-enriched coconut water after exercise-induced dehydration Southeast Asian J Trop Med Public Health 2007 38 4 769 85 17883020

[B18] Idárraga AP Aragón-Vargas LF Post-exercise rehydration with coconut water [abstract] Med Sci Sports Exerc 2010 42 5 575

[B19] Moran DS Heled Margaliot M Shani Y Laor A Margaliot S Bickes E Shapiro Y Hydration status measurement by radio frequency absorptiometry in young athletes - a new method and preliminary results Physiol Meas 2004 25 51 59 10.1088/0967-3334/25/1/005 15005304

[B20] Antonio J Sanders MS Ehler LA Uelmen J Raether JB Stout JR Effects of exercise training and amino acid supplementation on body composition and physical performance in untrained women Nutrition 2000 16 1043 1046 10.1016/S0899-9007(00)00434-2 11118822

[B21] Cheuvront SN Ely BR Kenefick RW Sawka MN Biological variation and diagnostic accuracy of dehydration assessment markers Am J Clin Nutr 2010 92 3 565 73 10.3945/ajcn.2010.29490 20631205

[B22] Wong SH Chen Y Effect of a carbohydrate-electrolyte beverage, lemon tea, or water on rehydration during short-term recovery from exercise Int J Sport Nutr Exerc Metab 2011 21 4 300 10 21813913 10.1123/ijsnem.21.4.300

[B23] Armstrong LE Maresh CM Castellani JW Bergeron MF Kenefick RW LaGasse KE Riebe D Urinary indices of hydration status Int J Sport Nutr 1994 4 3 265 79 7987361 10.1123/ijsn.4.3.265

[B24] Popowski LA Oppliger RA Patrick Lambert G Johnson RF Kim Johnson A Gisolf CV Blood and urinary measures of hydration status during progressive acute dehydration Med Sci Sports Exerc 2001 33 5 747 53 11323543 10.1097/00005768-200105000-00011

[B25] Lopez RM Casa DJ Jensen KA Demartini JK Pagnotta KD Ruiz RC Roti MW Stearns RL Armstrong LE Maresh CM Examining the influence of hydration status on physiological responses and running speed during trail running in the heat with controlled exercise intensity J Strength Cond Res 2011 25 11 2944 54 10.1519/JSC.0b013e318231a6c8 22024610

[B26] Spaccarotella KJ Andzel WD Building a beverage for recovery from endurance activity: a review J Strength Cond Res 2011 25 11 3198 204 10.1519/JSC.0b013e318212e52f 21993044

